# Methylmalonyl-CoA Epimerase Deficiency Mimicking Propionic Aciduria

**DOI:** 10.3390/ijms18112294

**Published:** 2017-11-01

**Authors:** Lenaig Abily-Donval, Stéphanie Torre, Aurélie Samson, Bénédicte Sudrié-Arnaud, Cécile Acquaviva, Anne-Marie Guerrot, Jean-François Benoist, Stéphane Marret, Soumeya Bekri, Abdellah Tebani

**Affiliations:** 1 Department of Neonatal Pediatrics and Intensive Care, Rouen University Hospital, 76000 Rouen, France; lenaig.abily-donval@chu-rouen.fr (L.A.-D.); Stephanie.Torre@chu-rouen.fr (S.T.); stephane.marret@chu-rouen.fr (S.M.); 2 Normandie Université, UNIROUEN, CHU Rouen, INSERM U1245, 76000 Rouen, France; soumeya.bekri@chu-rouen.fr; 3 Department of Metabolic Biochemistry, Rouen University Hospital, 76000 Rouen, France; samson.aurelie@gmail.com (A.S.); B.Sudrie-Arnaud@chu-rouen.fr (B.S.-A.); 4 Service Maladies Héréditaires du Métabolisme, Centre de Biologie et Pathologie Est, Centre Hospitalier Universitaire de Lyon et UMR, 69677 Bron, France; cecile.acquaviva-bourdain@chu-lyon.fr; 5 Department of Genetics, Rouen University Hospital, 76000 Rouen, France; Anne-Marie.Guerrot@chu-rouen.fr; 6 Hormonology and Biochemistry Department, Robert Debré Hospital, AP-HP, 75019 Paris, France; jean-francois.benoist@aphp.fr

**Keywords:** methylmalonyl-CoA epimerase, methylmalonic aciduria, propionic aciduria

## Abstract

Methylmalonyl-CoA epimerase (MCE) converts d-methylmalonyl-CoA epimer to l-methylmalonyl-CoA epimer in the propionyl-CoA to succinyl-CoA pathway. Only seven cases of MCE deficiency have been described. In two cases, MCE deficiency was combined with sepiapterin reductase deficiency. The reported clinical pictures of isolated MCE are variable, with two asymptomatic patients and two other patients presenting with metabolic acidosis attacks. For combined MCE and sepiapterin reductase deficiency, the clinical picture is dominated by neurologic alterations. We report isolated MCE deficiency in a boy who presented at five years of age with acute metabolic acidosis. Metabolic investigations were consistent with propionic aciduria (PA). Unexpectedly, propionyl-CoA carboxylase activity was within the reference range. Afterward, apparently intermittent and mild excretion of methylmalonic acid (MMA) was discovered. Methylmalonic pathway gene set analysis using the next-generation sequencing approach allowed identification of the common homozygous nonsense pathogenic variant (c.139C > T-p.Arg47*) in the methylmalonyl-CoA epimerase gene (*MCEE*). Additional cases of MCE deficiency may help provide better insight regarding the clinical impact of this rare condition. MCE deficiency could be considered a cause of mild and intermittent increases in methylmalonic acid.

## 1. Introduction

**Figure 1:**
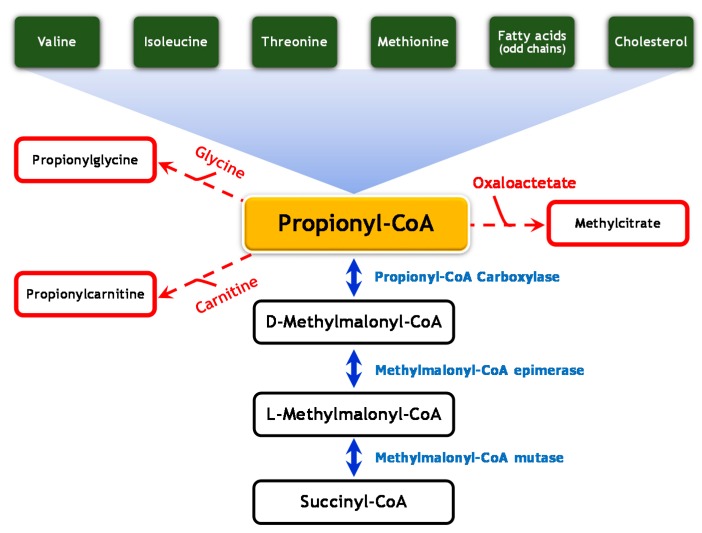
Metabolism of propionic acid. The catabolism of several amino acids (valine, isoleucine, threonine, and methionine) as well as odd chain fatty acids and cholesterol leads to the production of propionyl-CoA. When propionyl-CoA accumulates, other components—such as methylcitrate (formed by condensation of propionyl-CoA with oxaloacetate), propionylglycine, and propionylcarnitine—are found in urine and blood.

Isolated methylmalonic aciduria (MMA) is mainly caused by methylmalonyl-CoA mutase or deficiency of its co-factor, adenosylcobalamin. MMA onset usually occurs during the neonatal period with clinical intoxication signs such as hypotonia, encephalopathy, seizures, vomiting, and failure to thrive. Recently, guidelines have been issued for their management [1]. Of note, occurrences of atypical methylmalonic aciduria due to methylmalonyl-CoA epimerase (MCE) deficiency are less frequent. MCE converts d-methylmalonyl-CoA epimer to l-methylmalonyl-CoA epimer, which is the substrate of methylmalonyl-CoA mutase (Figure 1) [2]. The clinical picture of this entity is variable, spanning from acute metabolic decompensation to no symptoms. However, few cases of MCE deficiency have been described to date [3,4,5,6,7]. We report a case of MCE deficiency presenting with acute metabolic acidosis and biochemical features of propionic aciduria.

## 2. Case Report

**Figure 2:**
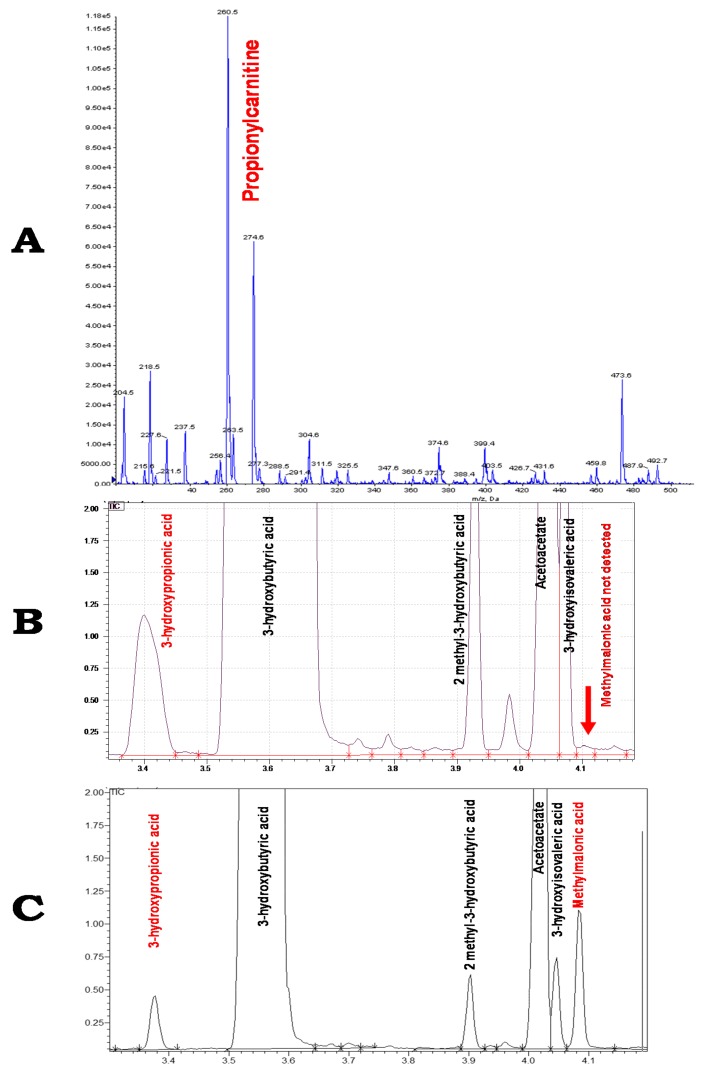
Acylcarnitine and urinary organic acid profiles. (**A**) Acylcarnitine profile using LC-MS/MS showing an elevation in propionylcarnitine following the acute metabolic crisis; (**B**) Acid organic profile using GC-MS presenting a high concentration of 3-hydroxypropionic acid with no methylmalonic acid detection; (**C**) Organic acid profile presenting a moderate increase of 3-hydroxypropionic acid and methylmalonic acid concentrations during follow-up, distant from any decompensation. 1.00e^4^ = 10,000; 1.00e^5^ = 100,000.

A boy was born to non-consanguineous parents with no specific family history. The birth was uneventful at 38 weeks of gestation. Motor development was normal, but the boy developed a moderate language delay. Frequent catabolism episodes with vomiting were noticed without decompensation. At five years of age, he presented with acute vomiting and feeding intolerance. Physical examination showed dehydration with severe signs such as tachycardia, polypnea, and hypotonia. Echocardiography and cerebral magnetic resonance imaging (MRI) results were normal. There was no kidney failure. Blood assessment revealed hydroelectrolyte balance alterations with metabolic acidosis (pH = 7.08, bicarbonates = 7.3 mmol/L, normal lactic acid, PCO_2_ = 25 mmHg) and hyperammmoniemia (100 µmol/L). Glycemia was normal. Ketone concentrations were high in urine samples. Blood count was unremarkable. Metabolic investigation results were consistent with propionic aciduria, with a large increase in plasmatic propionylcarnitine (17.5 µmol/L; N < 0.75), undetectable methylmalonylcarnitine (Figure 2A), and high urinary excretion of 3-hydroxypropionate (Figure 2B) and methylcitrate. Unexpectedly, propionyl-CoA carboxylase activity was within the reference range after assessment using cultured fibroblasts. Urinary organic acids analyses results determined apparently intermittent and mild excretion of methylmalonic acid. Fifteen assessments were performed, with results ranging from undetectable (once during the initial decompensation) to 212 µmol/mmol creatinine, with a mean of 69 and a median of 44 (Figure 2C). Increased urinary methylmalonic acid steered the diagnosis toward methylmalonic aciduria. Propionylcarnitine was constantly elevated in plasmatic acylcarnitine profiles, whereas traces of methylmalonylcarnitine were detected once. The plasmatic glycine concentration was within the reference range. Methylmalonic pathway gene set analysis using the next-generation sequencing (NGS) approach allowed identification of a homozygous nonsense variant (c.139C > T-p.R47*) in the methylmalonyl-CoA epimerase gene (MCEE–NM_032601.3). A hypoprotidic diet (1 g/kg/day) was introduced, as well as carnitine and vitamin B12 supplementation to enhance MMA metabolism. During follow-up, moderate language and attentional difficulties were noticed; however, the patient had satisfactory academic results in school.

## 3. Discussion

The diagnosis of MCE deficiency may be very challenging. In our case, the initial biochemical and clinical features were consistent with propionic aciduria (PA). Methylmalonic acid urinary excretion was apparently intermittent and mild and was not observed during the decompensation episode. Of note, the absence of MMA may be due to the lack of sensitivity of the organic acid assessment using gas chromatography/mass spectrometry. This drawback may be circumvented by using dedicated sensitive methods for MMA such as stable isotope dilution. NGS analysis of methylmalonic pathway genes allowed confirmation of MCE deficiency. MCE was described by Mazumder et al. in 1961 [2] as an enzyme converting d-methylmalonyl-CoA epimer to l-methylmalonyl-CoA epimer in the propionyl-CoA to succinyl-CoA pathway. The human gene *MCEE*, which codes for methylmalonyl CoA epimerase, was identified in 2001 [8]. Only seven cases of MCE deficiency have been described to date (Table 1), and in two cases MCE deficiency was combined with sepiapterin reductase deficiency [3,6]. The variant c.139C > T-p.R47* is frequent and was found during the homozygous state in six cases (including this present case) and during the heterozygous state in one case [7]. Concerning isolated MCE deficiency, Dobson et al. described the first two patients in 2006 [4]. The proband (P1) failed to thrive and was treated for severe gastro-oesophageal reflux. He presented at 13.5 months with severe metabolic acidosis. Organic urinary acids revealed methylmalonic and methylcitric acid elevation. This patient was treated with vitamin B12 infusions and was placed on a normoproteic diet. At age 12.5 years, growth and psychomotor development were satisfactory. His older sister (P2) presented with hydrocephalus at one year of age. After surgical treatment, development was normal. The urinary concentration of methylmalonic acid was elevated (95 µmol/mmol creatinine); therefore, a protein-restricted diet and sporadic vitamin B12 treatment were undertaken [4]. Isolated MCE deficiency was not associated with neurological impairment in patients P1, P2, P3, P5, and P6 (our case) (Table 1), who had MCE deficiency combined with sepiapterin reductase (SPR) deficiency. However, P7 and P8 also had neurological deterioration. These neurological features were related to SPR deficiency and improved with L-DOPA and 5HTP treatment [6]. Grandinger et al. described a three-year-old patient (P4) with dysarthria, mild spastic paraparesis, and ataxia related to isolated MCE deficiency; however, they did not report the *SPR* molecular study findings to confirm the exclusive MCE association with the neurological alterations [5].

## 4. Conclusions

A diagnosis of MCE is quite difficult to achieve because those with asymptomatic cases (P2 and P3) and those with severe acute metabolic acidosis cases have been described (P1, P5, and P6). Our case is intriguing because the initial presentation was consistent with PA. However, the continuing metabolic follow-up led to observations of moderate and intermittent elevations of MMA. Of note, elevated concentrations of propionylcarnitine with no increase in methylmalonylcarnitine do not allow for MMA to be excluded. In conclusion, this novel case contributes to the characterization of this rare condition and may help to elucidate intermittent elevations in methylmalonic acid.

## Figures and Tables

**Table 1:** Clinical, biochemical, and molecular characteristics of the described MCE patients.

Patient	Isolated MCE	Age at Diagnosis	Acute Metabolic Acidosis	Psychomotor Development	Language Development	Neurological Impairment	Urinary MMA Concentration (µmol/mmol Creatinine)	*MCEE* Variants		Reference
								Allele 1	Allele 2	
P1	Yes	13.5 months	Yes	Normal	Normal	No	180–1456	c.139C > T-p.Arg47*	c.139C > T-p.Arg47*	[4]
P2	Yes	14 years (patient 1 sibling) Asymptomatic	No	Normal	Normal	No	95–166	c.139C > T-p.Arg47*	c.139C > T-p.Arg47*	[4]
P3	Yes	Asymptomatic (patient 8 sibling)	No	Normal	Normal	No	1400	c.139C > T-p.Arg47*	c.139C > T-p.Arg47*	[5]
P4	Yes (?)	3 years	NC	Deteriorated motor function	Dysarthria	Mild spastic paraparesis Ataxia	621	c.178A > C-p.Lys60Gln	c.178A > C-p.Lys60Gln	[5]
P5	Yes	5 years	Yes	Normal	Normal	No	47–151	c.139C > T-p.Arg47*	c.379–644A > G-p.(?)	[7]
P6	Yes	5 years	Yes	Attentional difficulties	Moderate delay	No	No MMA excretion during the acute metabolic acidosis episode; afterwards 18-212	c.139C > T-p.Arg47*	c.139C > T-p.Arg47*	This study
P7	Combined with sepiapterin reductase deficiency	2 years	No	Retardation	NC	Spasticity	142	c.139C > T-p.Arg47*	c.139C > T-p.Arg47*	[3]
P8	Combined with sepiapterin reductase deficiency	1 month	No	Retardation	Limited speech	Axial hypotonia Postural instability Oculogyric crisis Fatigability with sleep disorders	60	c.139C > T-p.Arg47*	c.139C > T-p.Arg47*	[6]

MCE, methylmalonyl-CoA epimerase; MMA, methylmalonic acid; NC, Non communicated. ?, Not confirmed.
